# Effects of antibiotic growth promoter and its natural alternative on poultry cecum ecosystem: an integrated analysis of gut microbiota and host expression

**DOI:** 10.3389/fmicb.2024.1492270

**Published:** 2024-12-02

**Authors:** Chengyao Peng, Mahdi Ghanbari, Ali May, Thomas Abeel

**Affiliations:** ^1^Delft Bioinformatics Lab, Delft University of Technology, Delft, Netherlands; ^2^dsm-firmenich, Animal Nutrition and Health R&D Center, Tulln, Austria; ^3^dsm-firmenich, Science and Research, Delft, Netherlands; ^4^Infectious Disease and Microbiome Program, Broad Institute of MIT and Harvard, Cambridge, MA, United States

**Keywords:** antibiotic growth promoter (AGP), phytogenic feed additive (PFA), poultry, cecum, gut microbiome, host expression, host-microbe interaction

## Abstract

**Background:**

In-feed antibiotic growth promoters (AGPs) have been a cornerstone in the livestock industry due to their role in enhancing growth and feed efficiency. However, concerns over antibiotic resistance have driven a shift away from AGPs toward natural alternatives. Despite the widespread use, the exact mechanisms of AGPs and alternatives are not fully understood. This necessitates holistic studies that investigate microbiota dynamics, host responses, and the interactions between these elements in the context of AGPs and alternative feed additives.

**Methods:**

In this study, we conducted a multifaceted investigation of how Bacitracin, a common AGP, and a natural alternative impact both cecum microbiota and host expression in chickens. In addition to univariate and static differential abundance and expression analyses, we employed multivariate and time-course analyses to study this problem. To reveal host-microbe interactions, we assessed their overall correspondence and identified treatment-specific pairs of species and host expressed genes that showed significant correlations over time.

**Results:**

Our analysis revealed that factors such as developmental age substantially impacted the cecum ecosystem more than feed additives. While feed additives significantly altered microbial compositions in the later stages, they did not significantly affect overall host gene expression. The differential expression indicated that with AGP administration, host transmembrane transporters and metallopeptidase activities were upregulated around day 21. Together with the modulated kininogen binding and phenylpyruvate tautomerase activity over time, this likely contributes to the growth-promoting effects of AGPs. The difference in responses between AGP and PFA supplementation suggests that these additives operate through distinct mechanisms.

**Conclusion:**

We investigated the impact of a common AGP and its natural alternative on poultry cecum ecosystem through an integrated analysis of both the microbiota and host responses. We found that AGP appears to enhance host nutrient utilization and modulate immune responses. The insights we gained are critical for identifying and developing effective AGP alternatives to advance sustainable livestock farming practices.

## 1 Introduction

Antibiotic growth promoters (AGPs) are in-feed antibiotics at a sub-therapeutic concentration. The discovery of their growth-promoting effect in the 1950s has led to their wide usage in livestock farming practices, especially in the poultry sector (Kirchhelle, 2018). Common classes of antibiotics used as growth promoters include macrolide, β-lactam, tetracycline, peptides, fluoroquinolones, and polymyxins (Roth et al., 2019). Bacitracin, for example, was one of the most used peptide antibiotic growth promoters for poultry. It has been shown in different studies to be able to reduce poultry mortality rate, lower feed conversion ratio, and increase weight gain (Wicker et al., 1977; Ferket et al., 2002; Izat et al., 1990; Crisol-Martínez et al., 2017). However, in the late '90s, a clear connection between the use of antibiotics in livestock farming and the development of antibiotic resistance was recognized (Singer et al., 2003; Khachatourians, 1998; Witte, 1998; McDermott et al., 2002). Since then, the industry has been searching for effective natural alternatives to AGPs. The promising alternative options include probiotics, prebiotics, and phytogenic feed additives (PFAs) (Rahman et al., 2022). PFAs, the alternative of focus in this study, are plant-derived feed additives that can also enhance production (Abdel-Moneim et al., 2020). The beneficial effects of PFAs are believed to be attributed to polyphenols, the main bioactive compounds, which possess natural antioxidant and antimicrobial properties (Gadde et al., 2017).

Understanding the exact modes of action is essential to develop potent alternatives for AGPs. However, despite being used for decades, the molecular mechanisms behind the observed growth-promoting effects remain unclear (Brown et al., 2017). Based on the current evidence, the proposed hypotheses can be primarily divided into microbiota-centric and host-centric ones (Rahman et al., 2022). When used as AGP, the dosages of antibiotics are typically lower than the minimum inhibitory concentration needed to suppress the growth of pathogens directly (Broom, 2017). Consequently, popular microbiota-centric hypotheses mainly focus on how AGPs alter the gut microbiota composition, potentially promoting beneficial microbial populations and indirectly controlling pathogenic bacteria (Smirnov et al., 2005; Wati et al., 2015). Alternatively, microbial compositional changes can reduce the nutrient demands of the microbial community, making more nutrients available for the host (Plata et al., 2022). Host-centric hypotheses propose that AGPs can enhance nutrient uptake by the host, for example, through a thinner polarized epithelium (Page, 2006). Although the industry has increasingly shifted away from AGP in favor of alternatives such as PFAs, these alternatives also suffer from a similar lack of understanding regarding their molecular mechanisms.

To understand the underlying mechanisms of AGPs, many previous work utilized traditional culture-based methods and microarrays to examine the poultry gut microbial and host response. More recently, an increasing number of studies have employed high-throughput sequencing methods to examine the effects of AGPs and their alternatives on poultry gut ecosystem (Danzeisen et al., 2011; Salaheen et al., 2017; Paul et al., 2022; Huang et al., 2018; Lin et al., 2013; Costa et al., 2017; Fibi-Smetana et al., 2021; Luo et al., 2021; Oladokun et al., 2022). However, most of these studies focus on either the microbiota or the host gut, lacking a holistic view of the problem. The potential differences in the response of digesta and mucosa-associated microbiota are frequently neglected, despite increasing evidence of their distinct compositional and functional roles (Borda-Molina et al., 2018). In addition, the temporal nature of the response and the multivariate nature of the data are often overlooked. For instance, some studies rely solely on cross-sectional data collected at a single time point. Frequently, even when data is collected from multiple time points, only static and univariate analysis methods, like differential abundance and expression analysis, are used to identify affected microbes or host genes.

Our study aims to systematically assess the impact of a common AGP and a PFA on the poultry cecum ecosystem across four critical development stages. To achieve an integrated understanding, we analyzed both the gut microbiota (in the digesta and mucosa content) and host gene expression. Beyond standard differential abundance and expression analysis, we employed multivariate and time-course analyses to address the complex, time-dependent nature of this problem. Furthermore, we examined overall correspondences between the two profiles to explore potential interactions between the gut microbiota and host gene expression in the cecum. Besides, we identified significantly correlated pairs with treatment-specific patterns over time. The insights gained from our results are crucial for discovering and developing effective AGP alternatives for sustainable livestock farming.

## 2 Materials and methods

### 2.1 Materials

The data for our study were obtained from a previously conducted controlled randomized trial on poultry (Segura-Wang et al., 2021). Briefly, ninety-six healthy one-day-old male broiler chickens (Ross 308) were randomly allocated into twelve pens, with eight birds per pen. The twelve pens were further randomized into three dietary treatment groups: Control, AGP, and PFA, each group comprising four replicates.

Throughout the experiment, all birds were fed a standard basal diet. The Control group received no additional feed supplements. From day 3 to 35, the AGP group received an in-feed supplementation of zinc-bactracin (ALBAC^®^ , Huvepharma, Belgium; 20 mg/kg). The PFA group was supplemented with a phytogenic feed additive (Digestarom^®^ DC Power, Biomin Holding GmbH, Austria; 150 mg/kg feed).

Birds were euthanized on days 3 (baseline), 14, 21, and 35 for sample collection. Each time, two birds per pen were sacrificed to obtain samples of cecum digesta content, mucosa content, and tissue from the host. The samples from the two birds from each pen were pooled to form a single sample. To profile the cecum microbiota compositions, DNA was extracted from the collected digesta and mucosa content using the QIAamp PowerFecal DNA Kit (Qiagen) following the manufacturer's instructions. Library preparation and metagenomic sequencing were performed by LGC Genomics GmbH, using 150 bp paired-end reads (Illumina NextSeq 500 V2). RNA was extracted and purified from the tissue samples with the RNeasy Plus Mini Kit (Qiagen, Germany). The concentration and purity of isolated RNAs were estimated on a NanoDrop 2000 spectrophotometer (Thermo Scientific, 442 USA) and RNA quality (RIN) was determined by using the 4200 Agilent TapeStation System (Agilent). RNA-Seq libraries were prepared with the NEBNext Ultra RNA Library Prep Kit for Illumina, which includes poly(A) enrichment. The library was sequenced on a Illumina NovaSeq S1 SR100 machine.

### 2.2 Methods

**Figure 1:**
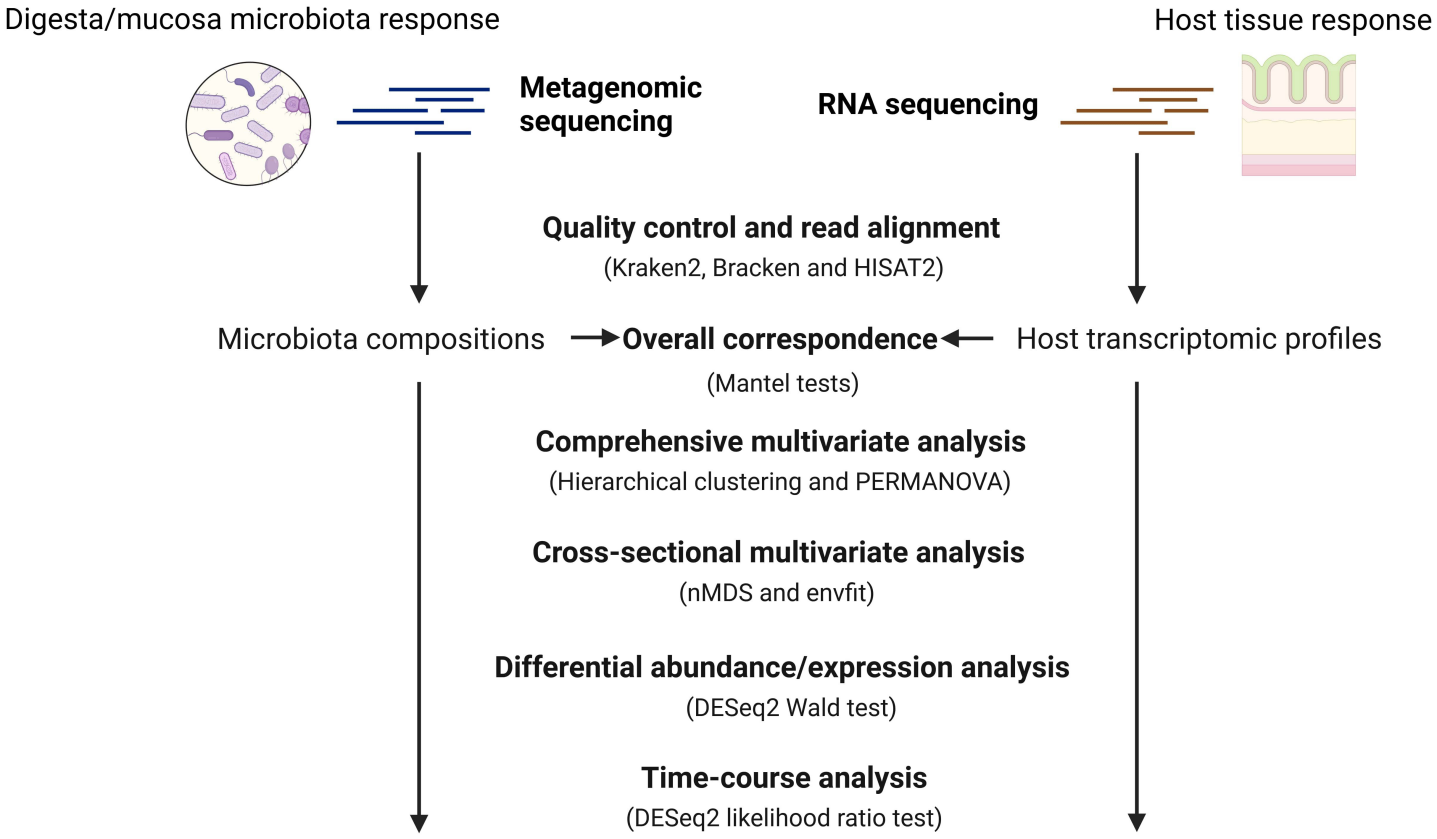
General workflow for the metagenomics and host transcriptomics analysis. To systematically assess the response of cecum ecosystem to AGP and PFA, we used metagenomic sequencing and RNA sequencing to profile the cecum microbiota compositions and host transcriptomics from chickens receiving different feed strategies at different ages. Later, we used Mantel tests to quantify the overall correspondence between the profiled microbiota compositions and the host gene expression. We used both the comprehensive and cross-sectional multivariate analysis to understand how different factors such as age, microbial sample type and feed additives impact our data. Later, differential abundance and expression analysis was performed to identify differentially abundant species and host expressed genes at each time point. In the end, a time-course analysis was conducted to find treatment-specific patterns in microbial species and host transcripts over time. Created with BioRender.com.

The overall workflow of this study can be found in Figure 1, while the details of each step and the methods used are described below.

#### 2.2.1 Quality control and read alignment

To ensure the quality of the metagenomic and host transcriptomic reads, we employed FastQC v0.11.7 (Andrews et al., 2017) for read quality assessment and Trimmomatic v0.39 (Bolger et al., 2014) for trimming low-quality bases.

For the metagenomic samples, Bowtie2 v2.3.5.1 (Langmead and Salzberg, 2012) and samtools v1.10 (Li et al., 2009) were used to remove the host sequences using the Gallus gallus genome GRCg6a downloaded from the NCBI FTP site (https://ftp.ncbi.nlm.nih.gov/genomes/all/GCF/000/002/315/GCF_000002315.6_GRCg6a/). Kraken2 v2.1.2 (Wood et al., 2019) was used to profile the taxonomic compositions of the samples at the species level using the GTDB release 207 (Parks et al., 2022) prebuilt by Struo2 (Youngblut and Ley, 2021). Bracken (Lu et al., 2017) was subsequently used to improve the accuracy of the abundance estimates generated by Kraken2. To reduce noise and spurious results, microbial species that have a relative abundance below 0.01% in <10% of the microbiota samples were excluded. In the following analysis of the microbiota, we separated the two sample types, digesta and mucosa, since they might react differently to changes in feed.

For the host transcriptomic samples, HISAT2 (Kim et al., 2019) and samtools v1.10 were used to map the transcriptomic reads to the Gallus gallus genome GRCg6a mentioned before. Next, featureCounts (Liao et al., 2014) was used to generate the read counts. Similarly, host transcripts representing <0.1% of all transcripts in fewer than 10% of the transcriptomic samples were removed.

#### 2.2.2 Overall correspondence between microbiota and host transcriptomic profiles

We converted the obtained digesta and mucosa microbiota compositions, as well as host transcriptomic profiles into distance matrices using Spearman's rank correlation to evaluate their overall correspondence, independently of the treatment groups. Subsequently, Mantel tests were conducted on each pair of the distance matrices to assess their correlations. This analysis was performed using the vegan R package v2.6.4 (Oksanen et al., 2022).

#### 2.2.3 Comprehensive multivariate analysis and statistical tests

In order to compare the influence of age, sample type and treatment on microbial compositions and host tissue expression, we employed hierarchical clustering to visualize and Permutational Multivariate Analysis of Variance (PERMANOVA) to quantify such influence of different sources.

Specifically, for all collected microbiota samples, we first computed the pairwise Bray-Curtis dissimilarity based on species-level relative abundance. Using this dissimilarity matrix, we visualized the underlying groupings of all microbiota samples by hierarchical clustering. To quantitatively assess the impact of various factors and their interactions on microbial community composition, we conducted Permutational Multivariate Analysis of Variance (PERMANOVA) test. The tested factors include the poultry age (four levels: Days 3, 14, 21, and 35), sample type (two levels: digesta and mucosa) and treatment (three levels: AGP, PFA and Control). We implemented the PERMANOVA test provided in the vegan R package v2.6.4. The associated p-values were derived by a permutation test with 1,000 iterations to assess the statistical significance. A significance level of 0.05 was used.

Similarly, we used hierarchical clustering and PERMANOVA to assess the impact of different sources on host expression. However, in this case, we transformed the raw counts by the regularized log function provided in DESeq2 R package v1.40.2 (Love et al., 2014) and used Euclidean distance to obtain the distance matrix for all transcriptome samples.

#### 2.2.4 Cross-sectional multivariate analysis

Dimension reduction and overlaying environmental variables onto the new ordination can provide valuable insights into how environmental factors impact a high-dimensional dataset. To evaluate such impact of the treatments on both data types at each single time point, we used non-metric multidimensional scaling (NMDS) implemented by the vegan R package v2.6.4 (Oksanen et al., 2022) to project the digesta and mucosa samples, as well as host transcriptomic samples collected at each single time point to a low-dimensional space. Specifically, NMDS was applied for microbial samples using the Bray-Curtis dissimilarity matrices, and host transcriptome samples using the Euclidean distance matrices. Subsequently, the treatment group variable was fitted to the new ordination plots using the *envfit* function (Clarke and Ainsworth, 1993) from vegan. The *envfit* function infers the influence of the treatments on the microbial compositions and host gene expression by examining the distribution and clustering of samples based on the treatment group variable within the ordination space. To quantify such influence, for each dataset at each time point, an *r*^2^ score was calculated by dividing the between-group variance by the total variance, indicating the proportion of the variance in the samples explained by the treatment group. Based on the ordination and the *r*^2^ score, an associated p-value was also provided by a permutation test with 1,000 iterations to assess the statistical significance. A significance level of 0.05 was used.

#### 2.2.5 Microbiota complexity and comparison

Traditionally, microbiota complexity has been assessed by different alpha diversity metrics. To evaluate the impact of the two feed additives on the microbiota complexity, we calculated Chao1 richness, Pielou's evenness and Shannon diversity for each microbiota sample using the Scikit-bio Python package v0.5.8 (Rideout et al., 2022). Later, Mann-Whitney *U*-Test was used to compare the three diversity metrics between each of the two treatment groups and the control group for both microbiota sample types (digesta and mucosa). To control for the risk of false positives, we corrected the resulting *p*-values from the Mann-Whitney *U*-Test by the Benjamini-Hochberg method. A significance level of 0.05 was used.

#### 2.2.6 Differential abundance and differential expression analysis

We utilized the Wald test in DESeq2 R package v1.40.2 to conduct differential abundance and expression analysis. DESeq2 is particularly advantageous for generating robust results when dealing with small sample sizes, thanks to its use of shrinkage for dispersion and fold change estimates (Baik et al., 2020). Though the method was originally developed for analyzing the RNA-seq data, its similarity to metagenomic data makes the tool also suitable for microbial count data. To be specific, DESeq2 was used to identify the differentially abundant species in both digesta and mucosa, as well as differentially expressed host genes. The analysis was performed separately at each four time points for each two feed additive groups. The species abundance and transcript expression level in the control group were used as the baseline for comparison. The log fold changes (LFC) were shrunk using the “apeglm” method to provide more accurate LFC estimates. The Wald test was then used to test if the LFC is equal to zero. The cutoff we used for the adjusted *p*-value was 0.05 and for the log_2_Fold Change (log_2_FC) was 1.

#### 2.2.7 Time-course analysis for microbial species and host transcripts

Apart from the differential abundance and expression analysis, we fully utilized the time-course experiment design by performing the likelihood ratio test (LRT) provided in DESeq2. This analysis allows the identification of treatment-specific changes over time. Similar to the Wald test, we compared the temporal trends of individual species abundance and host transcript expression in the AGP and PFA groups separately to those in the control group. Specifically, the full model design matrix contained the age factor, the treatment factor, and the interaction of the two (design = ~treatment+age+treatment:age). The reduced model removed the interaction term (design = ~treatment+age). DESeq2 fitted both the full and reduced models to the count data of a specific species or transcript using a generalized linear model (GLM) framework with negative binomial distribution. LRT tests were later used to test the statistical significance of the increase in the log-likelihood from the additional coefficient of the interaction term to identify treatment-specific changes over time.

Furthermore, to reveal possible interactions between the identified host transcripts and microbial species that have treatment-specific patterns, we calculated their pairwise correlations over time using Spearman's rank correlation and obtained the associated p-values by using the scipy Python package v1.10.1. Multiple-testing correction was used later on the p-values using Benjamini-Hochberg method.

#### 2.2.8 Gene ontology enrichment analysis for host transcripts

To understand the involved molecular functions of the identified differentially expressed host genes and host genes that show treatment-specific patterns, Gene Ontology (GO) enrichment analysis was performed using g:profiler (Raudvere et al., 2019). The default significance method g:SCS and the default cutoff value 0.05 were used.

## 3 Results

### 3.1 Age and sample type impact the cecum ecosystem more than the feed additive

To compare the effects of the feed additive and other factors on the cecum ecosystem, we profiled the collected microbiota samples and host tissue transcriptome samples. Hierarchical clustering and PERMANOVA were then used to visualize and quantify the impact of different factors on these profiles. Our results indicate that poultry age has a greater impact on both gut microbiota composition and host gene expression than feed additives. Moreover, within the gut microbiota, the sample type (digesta vs. mucosa) has a more significant impact on the microbial compositions than the feeding strategies.

**Figure 2:**
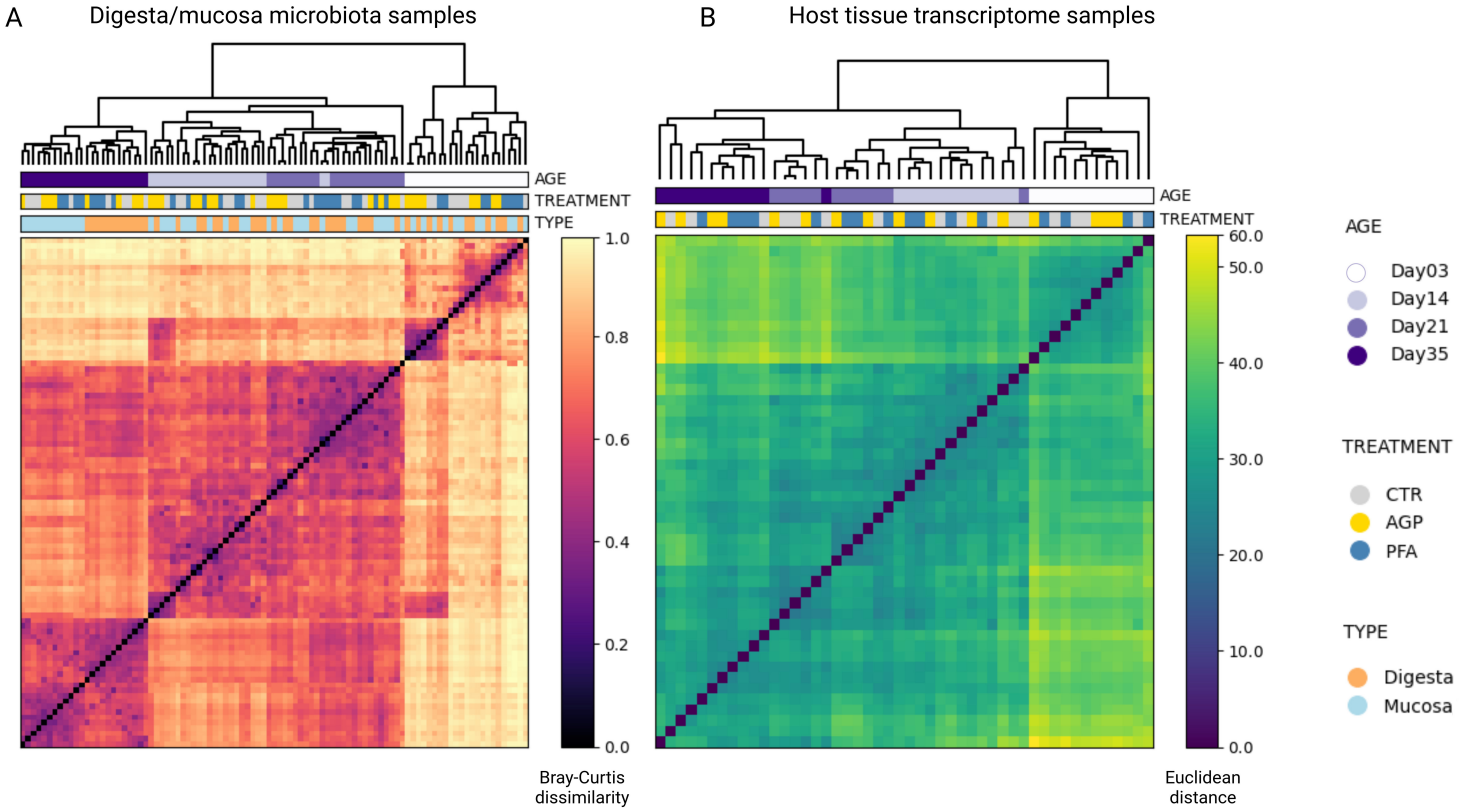
Visualizing the underlying grouping of the microbiota samples and host tissue transcriptome samples using hierarchical clustering. **(A)** Digesta and mucosa microbial samples. **(B)** Host tissue transcriptome samples. In both heatmaps, each row and each column is a sample, represented by its Bray-Curtis distances to other samples in the case of microbiota and Euclidean distances in the case of host tissue transcriptome. The metadata variables, age, treatment, and sample type, are mapped to each sample to reveal their relationships with the sample grouping.

Specifically, we taxonomically profiled the microbiota samples at the species level. Following quality control, read alignment and low-abundance filtering, we identified a total of 597 species. An overview of the ten most abundant species in digesta and mucosa content at each of the four time points is plotted in Supplementary Figure S1. Using hierarchical clustering, based on the Bray-Curtis dissimilarity matrix from all microbiota samples, we revealed that the microbial samples collected at the same age exhibited similar compositions and tended to cluster together (Figure 2A). On day 35, clear clustering was observed based on the sample type (digesta vs. mucosa), though no distinct treatment-related clusters emerged. Phylum-level analysis (Supplementary Figure S2) corroborated these findings, demonstrating significant temporal variations and consistent differences between the two microbial sample types, even at a high taxonomic level.

For the host tissue samples, after undergoing similar quality control and filtering procedures, we obtained 17,383 host transcripts. Similarly, hierarchical clustering using the Euclidean distance matrix also showed that age was the predominant factor influencing the transcriptome profiles (Figure 2B). In contrast, treatment did not significantly alter the clustering pattern within age-defined sub-clusters.

To further quantify the effects of various factors and their interactions on the cecum ecosystem, we conducted PERMANOVA tests (Table 1). As listed, PERMANOVA tests indicate that age, sample type and treatment all significantly influenced the microbial compositions. Specifically, age was the most significant factor, explaining 38% of the variation in the species-level compositions. Sample type and treatment also had significant effects, though to a lesser extent, accounting for 5.2 and 2.75% of the variation, respectively. Among the interaction terms, only the interaction between age and treatment was significant, explaining 6.07% of the variation. As for the host transcriptome profiles, PERMANOVA results showed that age was a highly significant factor, accounting for 32.6% of the variations. In contrast, treatment did not have a significant impact on the host gene expression and the interaction between age and treatment was also not significant.

**Table 1:** PERMANOVA results for the effects of age, sample type, treatment, and their interactions on gut microbiota compositions and host transcriptome profiles.

	**Term**	**Df**	**SS**	***R* ^2^**	***F*-value**	**Pr (>F)**
Gut microbiota	Age	3	9.962	0.380	20.439	<0.001***
	Type	1	1.364	0.052	8.395	<0.001***
	Treatment	2	0.722	0.028	2.221	0.002**
	Age × type	3	0.675	0.026	1.385	0.076
	Age × treatment	6	1.592	0.061	1.633	0.004**
	Type × treatment	2	0.143	0.005	0.440	1.000
	Age × type × treatment	6	0.221	0.008	0.226	1.000
	Residual	71	11.536	0.440		
	Total	94	26.214	1.000		
Host transcriptome	Age	3	9252	0.326	7.134	<0.001***
	Treatment	2	924	0.033	1.069	0.316
	Age × treatment	6	2,665.4	0.094	1.028	0.392
	Residual	36	15,563	0.548		
	Total	47	28,404.5	1		

The factors or variables being tested for gut microbiota and host transcriptome were listed with their degrees of freedom (Df). The Sum of Squares (SS) indicates the total variation attributable to each term in the model. *R*^2^ measures the proportion of total variance in the data that is explained by each term. The *F*-value indicates the ratio of the between-group variance to the within-group variance with respect to each term. Pr(>F) represents the *p*-value associated with the *F*-value. Significance levels are denoted by asterisks: ^*^p ≤ 0.05, ^**^p ≤ 0.01, ^***^p ≤ 0.001, with all significant values highlighted in bold.

### 3.2 Feed additives significantly influence the microbiota composition on later days

To examine the impact of feed additives on the cecum ecosystem at each single time point, we constructed 12 dissimilarity matrices for each combination of data sources (digesta microbiota, mucosa microbiota, host transcriptome) and time points. We then applied dimensionality reduction to these matrices and mapped the feed additive group onto the new ordination. Our analysis revealed that feed additives significantly influence the species-level microbiota compositions, particularly at the later stages of the experiments.

**Figure 3:**
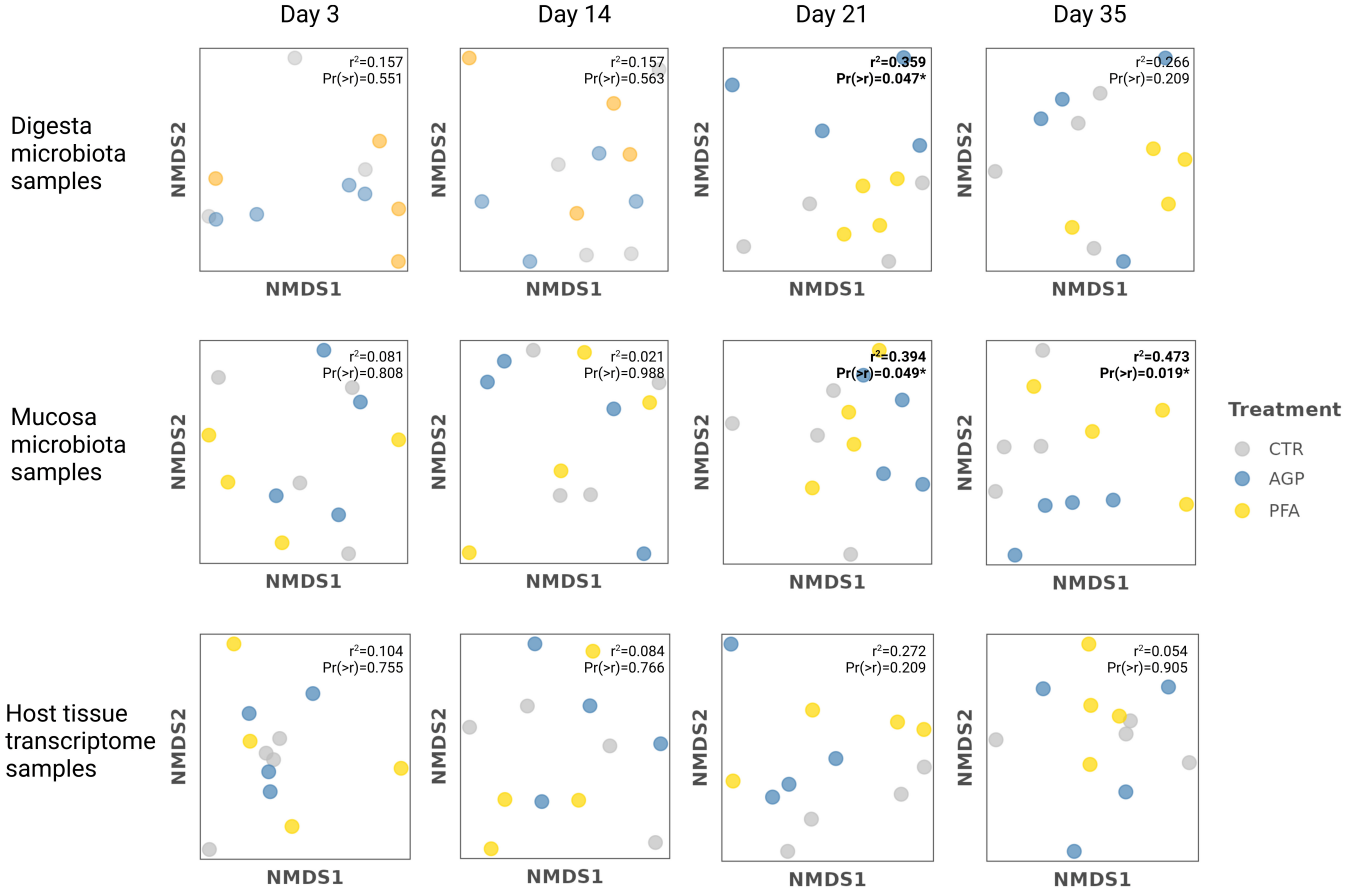
The NMDS ordinations of microbiota samples and host tissue samples at each time and the results from environmental fitting test analysis (envfit). The dissimilarity matrices generated from the digesta/mucosa microbiota data and the host transcriptome data were used for dimension reduction by nonmetric multidimensional scaling (NMDS). Based on that, environmental fitting test analysis (envfit) was applied to fit the treatment group onto the NMDS ordination. In this step, a *r*^2^ score was calculated to quantify the proportion of the variance in the microbial compositions explained by the treatment group. A corresponding p-value was also provided to indicate the statistical significance of the correlation between treatment group and ordination axes. Significance levels are denoted by asterisks: *p ≤ 0.05, with all significant values highlighted in bold.

To illustrate the extent to which the feed additives shaped the microbial community, we visualized the samples from each data source at each time point within the reduced-dimensional space. We quantified such influence by the proportion of variance in the microbial compositions explained by the treatment group and evaluated its statistical significance. As shown in Figure 3, feed additives significantly influenced the microbiota compositions primarily at the later stage of the experiment. High *r*^2^ scores and statistically significant p-values (*p* < 0.05) were observed in the microbial samples collected on day 21 and day 35. Specifically, the treatment group explained 35.9 and 39.4% of the variance in digesta and mucosa microbiota samples collected from day 21, respectively. On day 35, the treatment group accounted for 47.3% of the variance in the mucosa microbiota samples. However, no significant effect of the treatment group was detected in the host tissue transcriptome samples.

Despite the significant correlations between the treatment group and the microbial samples ordination in later days, we did not observe significant differences in microbial community complexity across any time point. Measures of microbial complexity, including Shannon diversity, Pielou's evenness, and Chao1 richness, remained consistent and did not differ significantly by the use of AGP or PFA at any time point. The detailed complexity indices calculated for all microbiome samples can be found in the Supplementary Table 1. As listed there, although not the primary focus of our study, we noticed that mucosa microbiota exhibited much lower richness than the digesta microbiota.

### 3.3 Up-regulated transmembrane transporter activity and metallopeptidase activity with antibiotic growth promoter administration

To identify individual microbial species and host transcripts with significantly different abundance or expression levels between the treatment groups, we performed differential abundance and expression analysis. Our results suggest that enhanced host mucosa transmembrane transporter activity is likely a key mode of action for antibiotic growth promoters.

In the differential abundance analysis of mucosa species, only two species showed significant changes across all time points: *Agathobaculum merdigallinarum* at day 14 and *Mediterraneibacter intestinipullorum* at day 21. Both species were significantly more abundant in the PFA group compared to the control group, with a log2 fold-change larger than 3.

**Figure 4:**
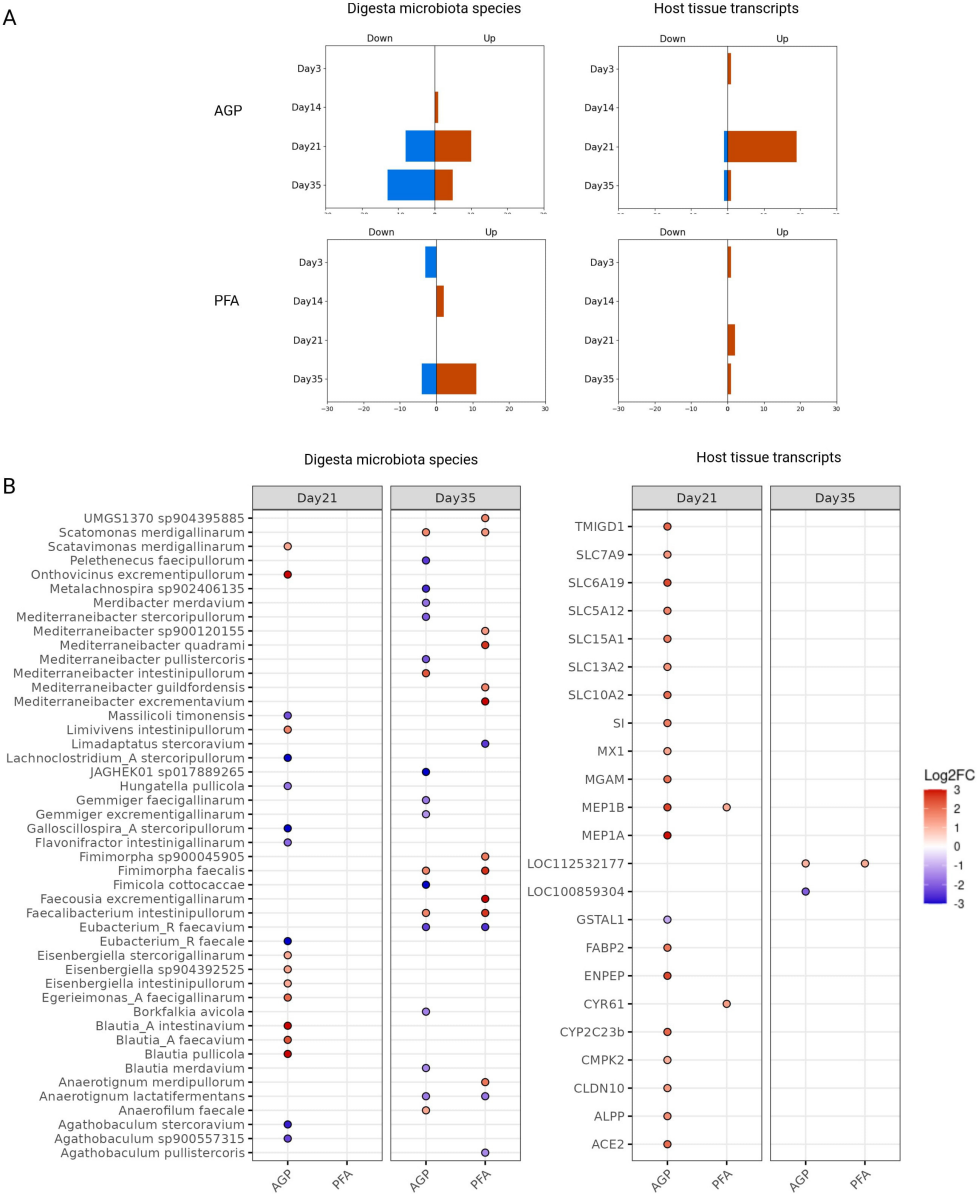
Differentially abundant cecum digesta species and host mucosa transcripts at each time point with each treatment. **(A)** The sum of the differentially abundant digesta species and expressed host transcripts. Their directions of fold-change were indicated by “down” and “up”. **(B)** The names of the identified digesta species and host mucosa transcripts that pass log_2_*FC* threshold (=1). For easier visualization, the values of log_2_*FC* were clamped to have the range between −3 and 3.

In contrast, a greater number of differentially abundant digesta species and expressed host genes were identified, indicating that the feed additives had a more substantial impact on them than the mucosa microbial species. Figure 4A shows the sum of these digesta species and expressed host genes for each treatment at each time point. As shown, there were few differentially abundant digesta species and host transcripts at day 3, suggesting that the broiler chicken had similar initial states before feed additive administration. Over time, we noted a gradual increase in the number of differentially abundant species and host transcripts, especially with AGP supplementation. Peaks for both digesta species and host mucosa transcripts were observed on day 21 in the AGP group, indicating a significant perturbation in the gut ecosystem at this time. Notably, the majority of differentially expressed genes at this time were up-regulated, including a group of solute carrier (*SLC*) genes from the solute carrier family. Figure 4B shows the detailed names and the log_2_*FC* values of the differentially abundant digesta species and host transcripts.

To characterize the response of host expression at day 21 to AGP, we performed a GO molecular function enrichment analysis, using the differentially expressed host genes at this time. The enriched terms in the GO context is shown in Supplementary Figure S3. The enrichment analysis showed that the key enriched molecular functions include secondary active transmembrane transporter activity (GO:0015291), carboxylic acid transmembrane transporter activity (GO:0046943), and metallopeptidase activity (GO:0008237), ranked by their statistical significance. These findings suggest that enhanced host mucosa transmembrane transporter activity and metallopeptidase activity may play a crucial role in the growth-promoting effects of the antibiotic growth promoter.

### 3.4 Modulated MIF and C1QBP expression over time with antibiotic growth promoter administration

In addition to the static differential abundance and expression analysis, we also modeled the temporal dynamics of microbial species and host gene expression levels. Using both a full and reduced model, we pinpointed the species and genes exhibiting AGP or PFA-specific changes over time through a likelihood ratio test. Our results indicate that AGP may also promote growth by modulating the expression of the Macrophage migration Inhibitory Factor (*MIF*) and Complement C1q Binding Protein (*C1QBP*) host genes in the cecum.

**Figure 5:**
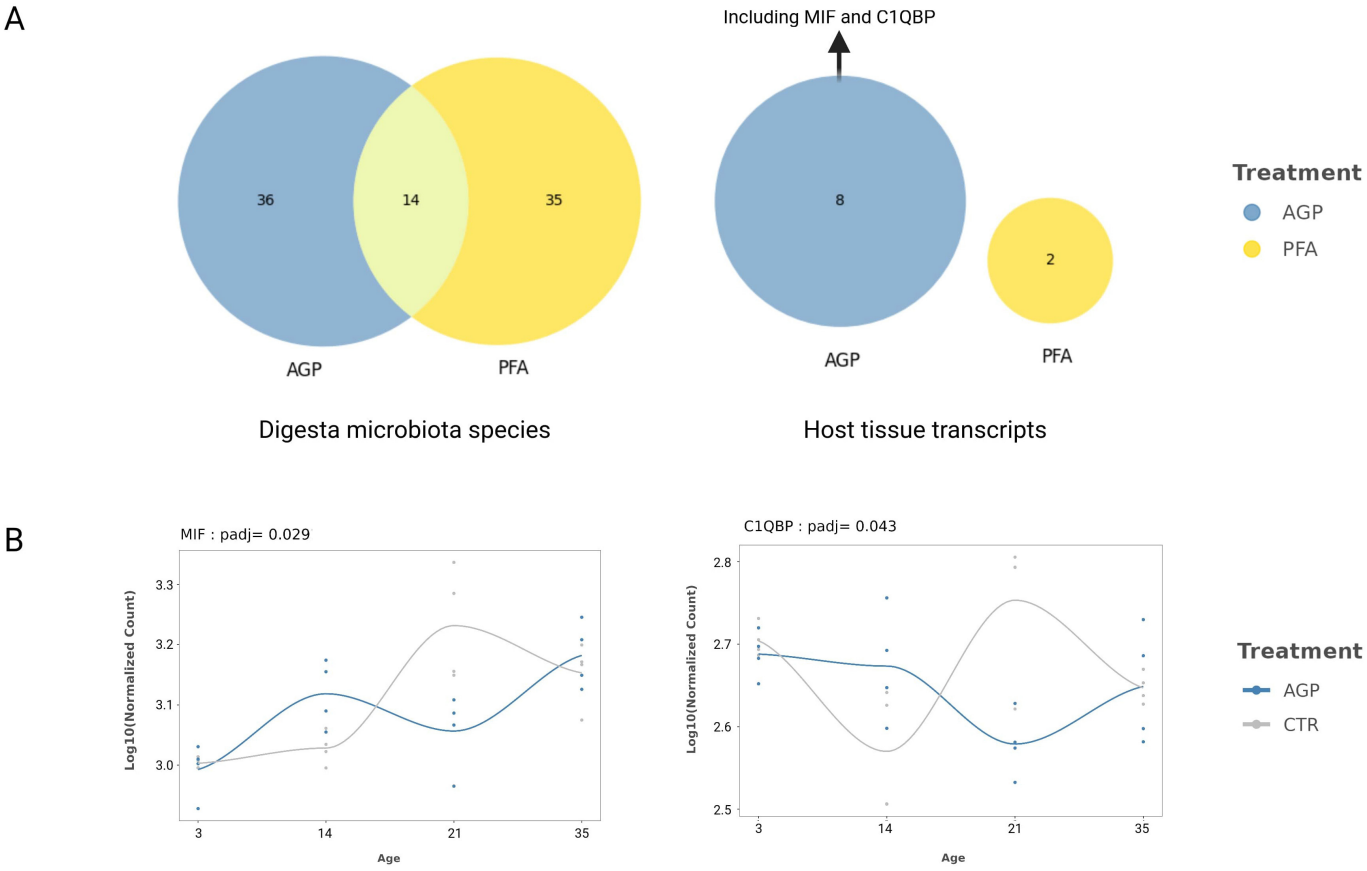
Identified species and host expressed genes that showed treatment-specific change across the time. **(A)** The number of shared and unique digesta species and host mucosa tissue transcripts that have treatment-specific changes over time. **(B)** The trends of MIF and C1QBP in the AGP and Control group. The lines were estimated using locally estimated scatterplot smoothing (LOESS).

Similar to the differential abundance and expression analysis, the time-course analysis revealed few significant findings for mucosal species, while identifying numerous digesta species and host expressed genes with treatment-specific alterations. Figure 5A provides an overview of these identified species and host transcripts for each treatment and the intersection between the two treatments. As shown, while AGP and PFA influenced the expression trajectory of 14 shared species across time, the sets of impacted host expressed genes were unique to each treatment. The complete table containing the names of the identified digesta species and host mucosa transcripts that have treatment-specific change can be found in the Supplementary Table 2.

We performed a GO molecular function enrichment analysis with the eight host transcripts identified in the AGP group. Given the well-studied role of MIF and C1QBP in the query gene set (Figure 5A), the significantly enriched terms include kininogen binding (GO:0030984) and phenylpyruvate tautomerase activity (GO:0050178). To visualize the change in the expression of these two genes in the AGP and control group, we plotted their trends using locally estimated scatterplot smoothing (LOESS) in Figure 5B. As shown, for both genes, the slops between all time intervals differ between the AGP and the control group, particularly after day 14.

**Figure 6:**
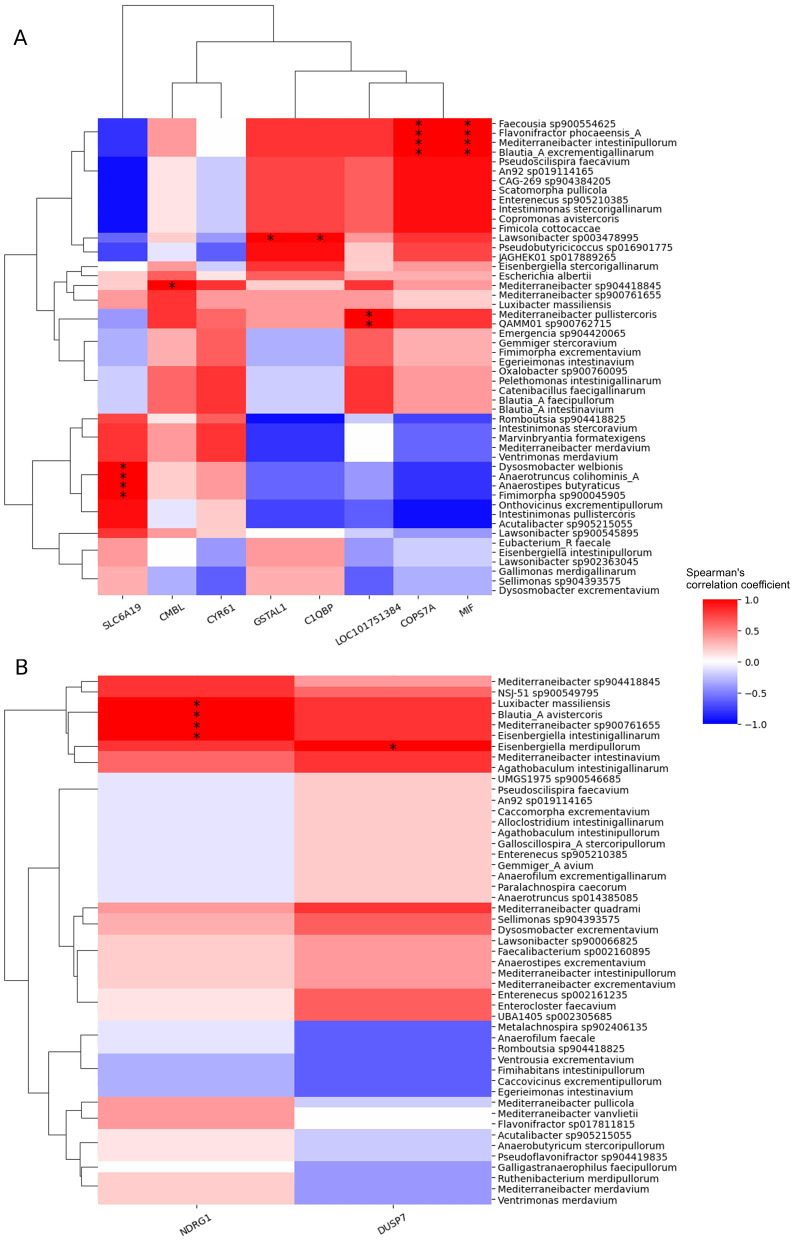
Spearman's correlation between digesta species and host transcripts with treatment-specific changes over time. **(A)** In the AGP group **(B)** In the PFA group The associated *p*-values were calculated by two-sided *t*-test and corrected using Benjamini/Hochberg method. The significant correlations are denoted by asterisks: *p ≤ 0.05, **p ≤ 0.01.

To reveal possible interactions between the identified host transcript and digesta species that showed different patterns for each feed additive, we calculated their pairwise correlation over time. Figure 6 illustrates the resulting Spearman's correlation coefficients for both treatment groups, with host transcripts on the *x*-axis and digesta species on the *y*-axis. Subsequent significance tests show several significant correlations between specific pairs of host transcript and digesta species. For instance, in the AGP group, the expression of host gene MIF exhibited a significant correlation with four species, *Faecousia sp900554625, Flavonifractor phocaeensis A, Mediterraneibacter intestinipullorum* and *Blautia A excrementigallinarum*. This suggests potential interactions between these genes and microbial species during the developmental stages of broiler chickens under AGP administration.

### 3.5 The host transcriptomics are positively correlated with gut microbiota profiles

To evaluate the overall correlation between host gene expression and microbiota composition, we performed Mantel tests. The results showed a very high Mantel statistic (*r* = 0.94) between the microbiota profiles from digesta and mucosa, indicating a strong positive correlation. This result is expected, given the close proximity of these microbial sample types. Interestingly, we also noted relatively high r values when comparing the distance matrix generated from host transcriptomics to the microbiota distance matrix (*r* = 0.72 with digesta microbiota and *r* = 0.73 with mucosa microbiota). These results suggest a positive relationship between the host mucosa transcriptomic profile and microbiota composition. In other words, the birds with similar cecum mucosa transcriptomic profiles tend to possess similar gut microbiota compositions. The significance of these correlations was confirmed by permutation tests, all showing very low *p*-values (*p* < 0.001). This indicates a high degree of consistency between host gene expression and microbiota compositions.

## 4 Discussion

In-feed antibiotic growth promoters have been used for decades to promote the health and growth of poultry. Despite their reproducible growth-promoting effects observed, the precise underlying mechanisms remain unclear. Common hypotheses regarding their mode of action are divided into microbiota-centric and host-centric perspectives. Our study systematically examined the impact of a common AGP and a natural alternative phytogenic feed additive on the cecum ecosystem during a critical period of poultry development. This included analyzing the digesta and mucosa microbiota, as well as the host gene expression.

Our comprehensive analysis of the cecum ecosystem revealed several key insights into the effects of feed additives and other influencing factors. While feed additives significantly impact gut microbiota compositions, they do not notably affect host gene expression, as evidenced by our PERMANOVA results. Instead, poultry age emerged as a more significant factor, exerting a greater influence on the cecum ecosystem than the feed additives. The observed significant interaction between age and treatment in microbiota samples indicates that the effects of feed additives on microbial communities vary with the age of the poultry. To further explore this, we analyzed the data by dividing the original dataset into four cross-sectional datasets based on age. We employed dimensionality reduction techniques to assess treatment effects at each time point. The results from the cross-sectional analysis supported our PERMANOVA findings, demonstrating that feed additives impact overall microbiota compositions more prominently than host gene expression, with significant effects in the later stages of development. The results we gained regarding the microbiota align with the findings from Zou et al. (2022), where sampling site and host age were found to have more substantial impact on the poultry microbiota than diet and AGPs. The same study also noted that the impact from diet and AGPs became more pronounced over time, suggesting that feed additives, such as AGPs, require a period to significantly influence microbial populations. Furthermore, our observation of no significant change with AGP supplementation in microbiota complexity is consistent with Kumar et al. (2018).

Later, we identified species and host genes significantly affected by the feed additive treatments using differential abundance and expression analyses. In the AGP group, we observed notable peaks in both digesta microbes and host gene expression on day 21, suggesting a substantial perturbation in the gut ecosystem at around this time. This finding aligns with our dimension reduction analysis results. Among the differentially abundant digesta microbes, we noted species from genera such as *Blautia, Eisenbergiella*, and *Agathobaculum*. While the role of these species in poultry is not well-studied, *Blautia*, in particular, is recognized for its potential probiotic effects (Liu et al., 2021). Further investigation into the functional roles of these species is necessary to understand their impact on gut health. Meanwhile at day 21, the majority of differentially abundance host genes were up-regulated, particularly those associated with secondary active transmembrane transporter activity, carboxylic acid transmembrane transporter activity and metallopeptidase activity. These enhanced molecular functions are important for the growth and health of the animals, and may contribute to the growth-promoting effects of AGPs. Specifically, secondary active and carboxylic acid transmembrane transporters encoded by the up-regulated *SLC* genes are able to facilitate the transport of essential nutrients and molecules across the membrane of epithelial cells for maintaining gut homeostasis, such as amino acids, short-chain fatty acids and bile acids (Oelkers et al., 1997; Pramod et al., 2013; Fotiadis et al., 2013; Hagenbuch and Dawson, 2004; Wright, 2013). Metallopeptidases encoded by up-regulated genes such as *ENPEP, MEP1A* and *MEP1B*, can catalyze the hydrolysis of peptide bonds. These enzymes play a role in tissue remodeling and repair, which may be a response to gut inflammation. For example, *MEP1A* and *MEP1B* encode meprin α and meprin β, respectively. In mice and humans, meprins are known to be able to protect the gut epithelium against toxic peptides and pathogenic bacteria (Vazeille et al., 2011; Werny et al., 2022). However, their specific functions in poultry cecum remain to be thoroughly characterized.

In the subsequent time-course analysis, we spotted dozens of digesta species and a few host expressed genes showing treatment-specific changes over time for the two treatments. Such host genes under AGP administration include *C1QB* and *MIF*. These genes were enriched in kininogen binding and phenylpyruvate tautomerase activity. Kininogens are precursors to kinins, which are involved in the inflammatory response and tissue repair (Couture et al., 2001). Encoded by the *MIF* gene, phenylpyruvate tautomerase facilitates the conversion of phenylpyruvate to phenylacetate (Rosengren et al., 1997). This enzyme is also implicated in cellular signaling pathways related to inflammation and immune responses (Calandra and Roger, 2003). The AGP-specific changes over time of these genes suggest that AGPs modulate immune responses through these pathways, which could contribute to their growth-promoting effects. In contrast, PFA application did not induce significant changes in the host gene expression profile over time. However, it did influence a comparable number of digesta species. Despite this, there was minimal overlap between the host genes and digesta species affected by both feed additives. This indicates that AGP and PFA influence the cecum ecosystem differently, suggesting distinct mechanisms of action for them.

Compared to digesta microbiota, we found a less significant response in mucosa microbiota, according to both the differential abundance analysis and time-course analysis. The lower richness we noticed in mucosa microbiota may partly explain such difference. Furthermore, due to the distinct environments, the mucosa region is generally more stable than the lumen in terms of nutrient availability (Borda-Molina et al., 2018), which likely buffers the mucosa microbiota from dietary changes, such as the introduction of feed additives.

In the end, to examine potential associations between the host expression and the microbiota, we applied Mantel tests using their overall profiles and calculated their pairwise association over time. Mantel tests showed a significant positive correlation between host transcriptomic profiles and gut microbiota profiles. This suggests that poultry with similar gut expression profiles tend to have similar gut microbial compositions. This strong correlation may be attributed to the feeding additives, which impact both host gene expression and microbiota composition. It may also reflect interactions between host expression and microbiota composition, consistent with previous studies (Nichols and Davenport, 2021). Notably, the time-course analysis identified 17 pairs of significant correlated digesta species and host transcripts with AGP-specific changes over time, indicating potential AGP-specific interactions.

In conclusion, our study provides a systematic evaluation of the impact of a common AGP and a PFA on the poultry cecum ecosystem at four key developmental stages. We found that while feed additives significantly affect microbiota composition at later stages, they have a minimal impact on the overall host gene expression. The distinct pattern of differential abundance and expression between the AGP and PFA highlight differences in their modes of action. Specifically, our findings suggest that AGPs may enhance nutrient utilization and modulate immune responses, contributing to their growth-promoting effects. These insights are critical for the development of effective AGP alternatives and for advancing sustainable practices in livestock farming.

## Data Availability

Publicly available datasets were analyzed in this study. This data can be found here: the metagenomic datasets used in this study are available in the NCBI BioProject under accession number PRJNA715658, while the host RNA-seq datasets can be found in NCBI BioProject PRJNA1147906. The intermediate data and code used in this paper can be found at: https://github.com/AbeelLab/poultry_cecum_response.
