# Supplementary material for: Effects of antibiotic growth promoter and its natural alternative on poultry cecum ecosystem: an integrated analysis of gut microbiota and host expression
Source: Front Microbiol. 2024 Dec 2;15:1492270. doi: 10.3389/fmicb.2024.1492270 (PMC11646981; doi:10.3389/fmicb.2024.1492270)
Supplement: Supplementary file 1 [file Data_Sheet_1.pdf]

Supplementary Material

1 SUPPLEMENTARY DATA

See supplementary Data Sheet 1 for the identified digesta microbial species and host expressed genes with AGP or PFA-specific effect over time.

2 SUPPLEMENTARY TABLES AND FIGURES

We visualized the relative abundance of all the samples (Fig S2) on the phylum-level and noticed that microbiome compositions went through drastic change over time with certain differences between the two sample types. Over the experiment time, for example, from day 3 to day 14, the proportion of *Firmicutes_A* significantly increased, accounting for approximately 90% at day 14 in terms of relative abundance in both digesta and mucosa microbiome. In contrast, the relative abundance of *Firmicutes*, *Actinobacteriota* and *Proteobacteria* decreased during this same early period. During day 21 to day 35, we observed a noticeable increase in the relative abundance of Bacteroidota, rising from nearly zero to approximately 10% to 20%. Although sample type does not influence phylum-level compositions as significantly as age, we still observed consistent differences between digesta and mucosa samples. For instance, digesta samples exhibited a relatively higher abundance of *Firmicutes_A* and a lower abundance of *Actinobacteriota* compared to mucosa samples from the same time points. However, such consistent pattern at phylum-level is not obvious for both feed additives.

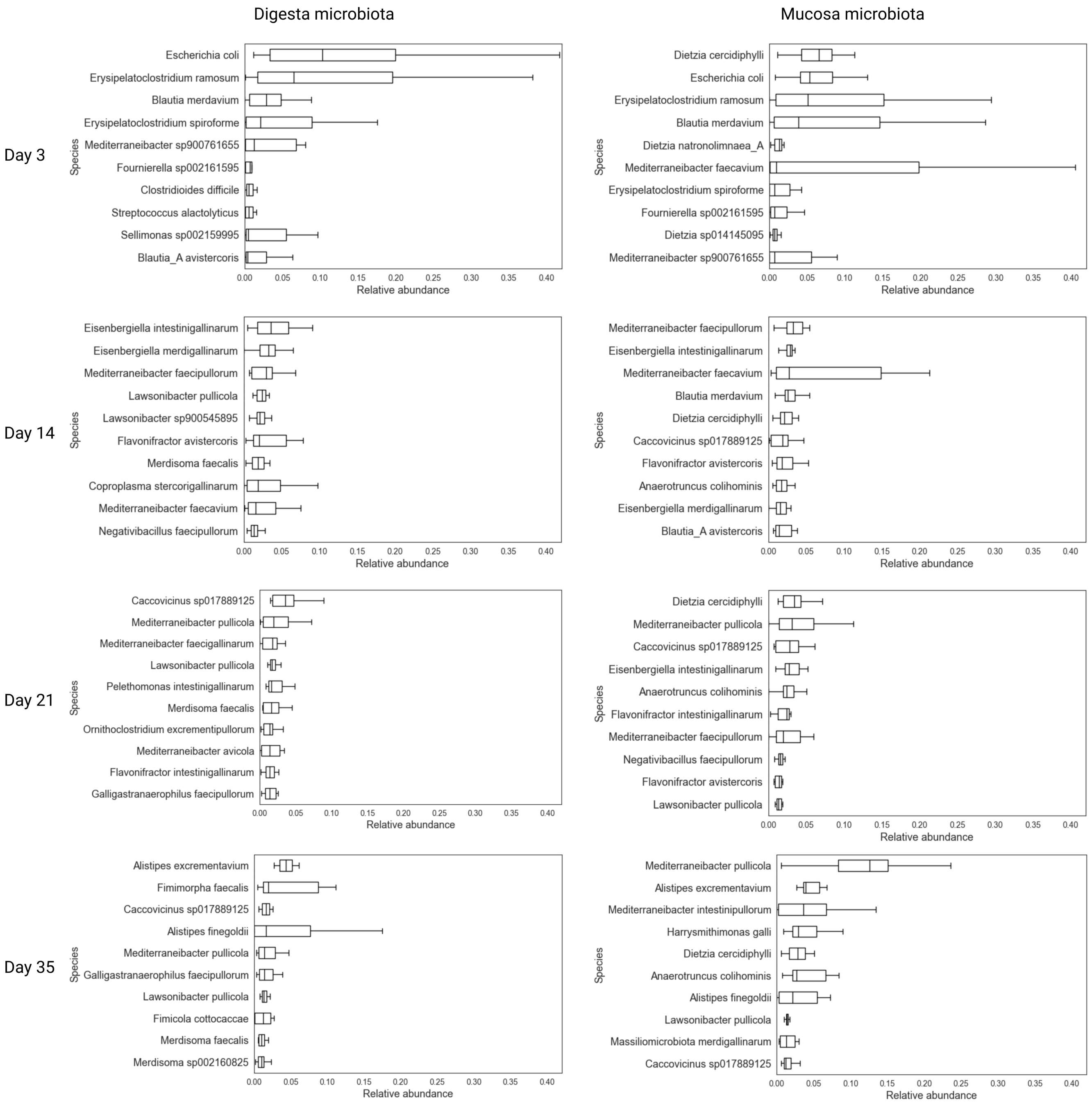

Figure S1. Relative abundance of ten most abundant species in digesta and mucosa content across the four time points. The x-axis shows the relative abundance and the y-axis displays the species names. The species were ranked by the median relative abundance across all specific type of samples collected at each time.

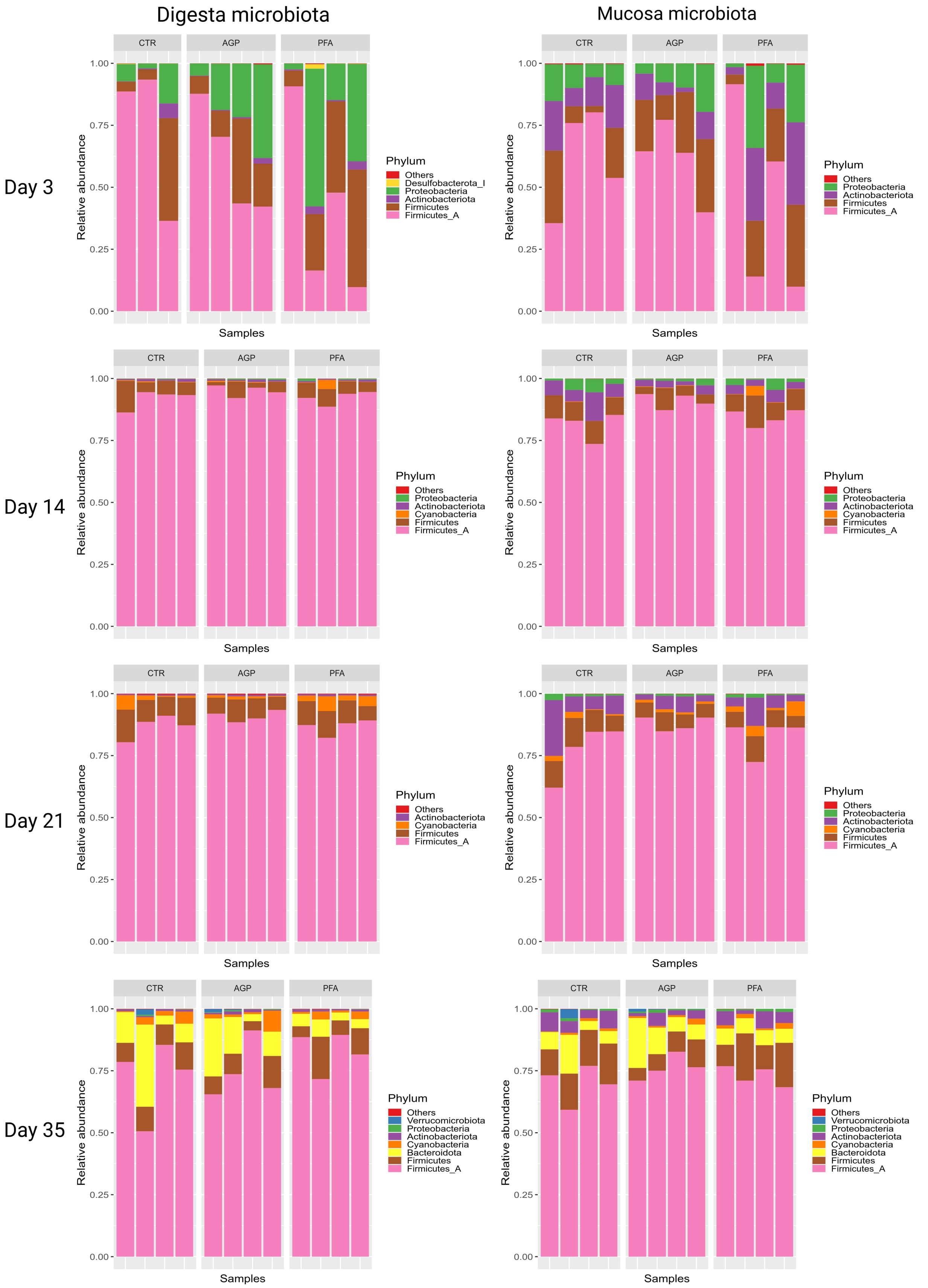

Figure S2. Overview of phylum-level compositions of profiled cecum microbome samples across four time points . The bars represent the relative abundance of reads assigned to different phylum, grouped by three treatment groups. Low-abundance phylum ($< 1\%$) were collapsed to improve the readability.

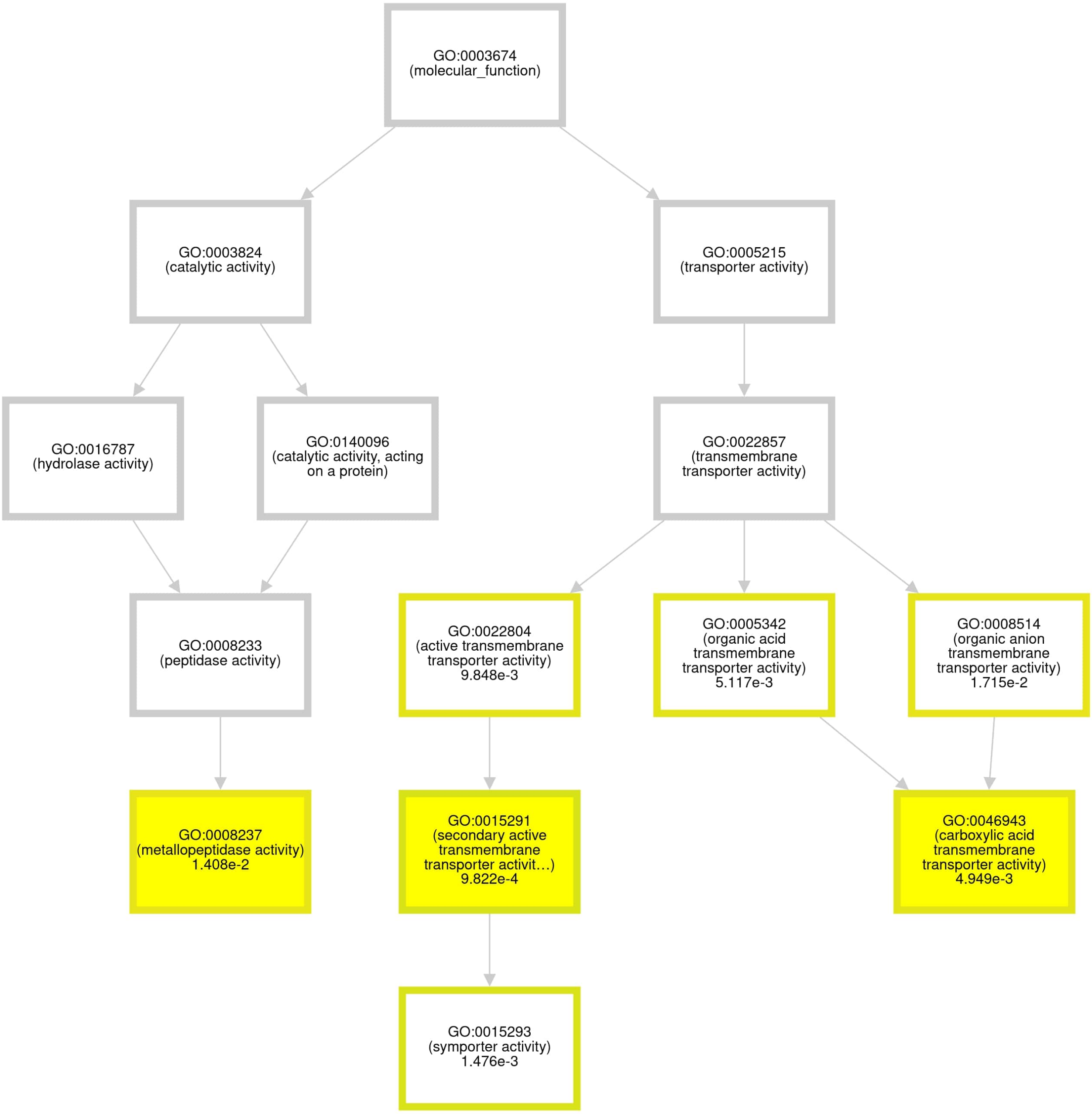

Figure S3. Enriched GO molecular function terms in GO context and their corresponding adjusted p-value by differentially expressed genes identified at day 21 in the AGP group. The significantly enriched terms are colored with a border that corresponds to their enrichment p-values. Highlighted terms with the yellow background are the driver terms.
